# CT Texture Analysis of Perihilar Cholangiocarcinoma—Associations With Tumor Grading, Tumor Markers and Clinical Outcome

**DOI:** 10.1002/cnr2.2132

**Published:** 2024-09-22

**Authors:** Jakob Leonhardi, Arsen Sabanov, Anne Kathrin Höhn, Robert Sucher, Daniel Seehofer, Matthias Mehdorn, Benedikt Schnarkowski, Sebastian Ebel, Timm Denecke, Hans‐Jonas Meyer

**Affiliations:** ^1^ Department of Diagnostic and Interventional Radiology University of Leipzig Medical Center Leipzig Germany; ^2^ Department of Surgery University of Leipzig Medical Center Leipzig Germany; ^3^ Department of Pathology University of Leipzig Medical Center Leipzig Germany; ^4^ Department of Surgery, Division of General, Visceral and Transplant Surgery Medical University of Graz Graz Austria

**Keywords:** computed tomography, extrahepatic cholangiocarcinoma, Klatskin tumor, texture analysis

## Abstract

**Background:**

Texture analysis derived from computed tomography (CT) may provide clinically relevant imaging biomarkers associated with tumor histopathology. Perihilar cholangiocarcinoma is a malignant disease with an overall poor prognosis.

**Aims:**

The present study sought to elucidate possible associations between texture features derived from CT images with grading, tumor markers, and survival in extrahepatic, perihilar cholangiocarcinomas tumors.

**Methods:**

This retrospective study included 22 patients (10 females, 45%) with a mean age of 71.8 ± 8.7 years. Texture analysis was performed using the free available Mazda software. All tumors were histopathologically confirmed. Survival and clinical parameters were used as primary study outcomes.

**Results:**

In discrimination analysis, “S(1,1)SumVarnc” was statistically significantly different between patients with long‐term survival and nonlong‐term survival (mean 275.8 ± 32.6 vs. 239.7 ± 26.0, *p* = 0.01). The first‐order parameter “skewness” was associated with the tumor marker “carcinoembryonic antigen” (CEA) (*r* = −0.7, *p* = 0.01). A statistically significant correlation of the texture parameter “S(5,0)SumVarnc” with tumor grading was identified (*r* = −0.6, *p* < 0.01). Several other texture features correlated with tumor markers CA‐19‐9 and AFP, as well as with T and N stage of tumors.

**Conclusion:**

Several texture features derived from CT images were associated with tumor characteristics and survival in patients with perihilar cholangiocarcinomas. CT texture features could be used as valuable novel imaging markers in clinical routine.

## 1 Introduction

Texture analysis, as an evolving tool of image analysis, allows the quantification of radiological images. This technique may be able to provide new imaging biomarkers [1, 2, 3]. In particular, computed tomography (CT) images are used to perform this technique and seem to be the most suitable for this type of analysis due to their robust image acquisition and high availability [1, 2, 3].

Texture analysis includes “first order features”, which are also known as histogram features, and “second order features.” In the category of the first order features, the image features are issued into histograms without concern for spatial relationships [1]. These parameters, among others comprising “skewness,” “kurtosis,” and “entropy,” have been already shown to reflect different histopathology parameters in several different tumor entities [3, 4, 5, 6]. Skewness is a measure for the asymmetry of the histogram, kurtosis for the peak of the histogram, and entropy of the heterogeneity of the histogram [3]. For “second order features,” the spatial relationships between voxels with similar gray levels are quantified with different methods. Therefore, texture features might provide a measure of intralesional heterogeneity. As higher heterogeneity of tumors has been shown to correlate with poorer patient survival [7], texture features as imaging biomarkers may prove to be more useful than the above‐mentioned histogram features.

Cholangiocarcinoma is the second most common hepatic primary malignancy after hepatocellular carcinoma and accounts for 3% of digestive cancer [8]. Cholangiocarcinomas are classified according to their anatomical subtype, which mainly are intrahepatic and extrahepatic cholangiocarcinomas. The latter are further subdivided into perihilar and distal cholangiocarcinomas [9]. Perihilar is the most common subtype in literature and represents 50%–67% of all cholangiocarcinomas [8]. This tumor entity has an overall poor prognosis and presents frequent recurrence after surgical treatment. Relapse‐free survival for extrahepatic cholangiocarcinomas is reported to be just up to 52% in the first 2 years and down to 38% after 5 years [10]. The median survival is nowadays ranging from only 19 to 39 months [11].

Because of these very aggressive characteristics, the clinical management still remains a challenge. Conventional staging is usually performed by cross‐sectional imaging using either CT or MRI scanners. Typical scanning protocols include contrast media administration and scanning in arterial and portal venous phase. The diagnostic sensitivity has been shown to range from 75% to 92% [12, 13]. However, conventional analysis of CT images can only provide information on potential resectability and tumor spread [14]. Better noninvasive characterization of cholangiocarcinoma may be critical for patient care. There are only few methods to obtain histopathology preoperatively through endoscopic methods or percutaneous biopsy. However, there is still some diagnostic uncertainty [9]. In these cases, CT texture analysis may provide further prognostic information that is helpful for more detailed staging and thus further clinical information.

This study hypothesizes that CT‐derived texture analysis correlates with biological characteristics of perihilar cholangiocarcinomas, including tumor grading, TNM staging, tumor marker levels, and postoperative patient survival. In that case, texture analysis could possibly represent a useful tool in clinical routine to further characterize these tumors noninvasively during the initial tumor staging and to even improve the clinical management.

## 2 Methods

### 2.1 Study Design

For this retrospective, observational study, all procedures performed involving human participants were in accordance with the ethical standards of the institutional and/or national research committee and with the 1964 Helsinki declaration and its later amendments or comparable ethical standards. It received ethical approval from the local ethics committee at the medical faculty of the university of Leipzig (EK: 243‐14‐1407‐2014). All participants provided written informed consent of this post hoc scientific analysis.

The radiological database of the University hospital of Leipzig was retrospectively screened between 2016 and 2022 for available and suitable imaging data of perihilar extrahepatic cholangiocarcinomas.

Inclusion criteria were sufficient presurgical CT data, laborchemical data, and performed surgical resection with histopathological examination and follow‐up survival data.

Overall, 22 patients (*n* = 10 females, 45%) with a mean age of 71.8 ± 8.8 years and histopathological proven perihilar cholangiocarcinoma were enrolled. The postoperative 30‐day mortality and long‐term survival was evaluated as primary study outcomes.

### 2.2 Surgical Treatment

All included patients underwent surgical treatment with curative intent. The used nomenclature of hepatic anatomy and resection was in compliance with the Brisbane 2000 classification system. A series of anatomic left and right hemihepatectomies was carried out with or without wedge resections of neighboring liver segments. In some of the cases, extended left and right hemihepatectomies commonly referred to as trisectionectomy were performed. En bloc resection of segment 1 was case dependent [10, 15, 16]. Roux‐en‐Y anastomosis was used to reconstruct the gastrointestinal tract. Furthermore, regional lymphadenectomie was performed in the lymph node groups 1, 2, and 3 [17]. Postoperative follow‐ups of the treated patients were performed in a clinical outpatient setting.

### 2.3 Histopathology and Laboratory Analyses

All resected perihilar cholangiocarcinomas were histopathologically analyzed blinded to the imaging results in clinical routine.

The tumor markers carcinoembryonic antigen (CEA) (available in *n* = 13 patients, 59.1%), carbohydrate antigen 19‐9 (CA 19‐9) (available in *n* = 14 cases, 63.6%), and alpha‐fetoprotein (AFP) (available in *n* = 8 of the cases, 36.4%) were determined before the surgical treatment in a clinical setting. In the missing cases, the diagnostic work‐up was performed in other hospitals without measuring tumor markers.

Preoperative bilirubine and hemostaseology of the serum were also assessed.

### 2.4 Image Analysis

CT imaging was performed in a clinical setting with a 256 slice CT scanner (iCT, Philips, Amsterdam, Netherlands) after intravenous application of 90 mL iodinated intravenous contrast medium (injected at a rate of 2 mL/s by a power injector [Medtron GmbH, Germany]), with a scan delay of 70 seconds after onset of injection. The portal‐venous phase images were evaluated for all patients. Typical imaging parameters were 120 kVp and 150–300 mAs. Reconstructed slice thickness was 1 mm.

### 2.5 Texture Analysis

CT images were stored in DICOM format. Further processing of the images was performed with the free available texture analysis software MaZda (version 4.7, available at http://www.eletel.p.lodz.pl/mazda/) [18, 19]. A polygonal region of interest (ROI) was placed on the representative axial slice with the largest tumor diameter. The ROI was drawn within the mass‐forming area of the tumor and it was clearly located within the lesion. The ROI was drawn by a trained resident with 5 years of general radiology experience (J.L.). In difficult cases, a board‐certified radiologist was consulted with 7 years of experience in hepatobiliary radiology (H.J.M.) to determine the exact tumor boundaries.

Images were analyzed in 1‐mm abdomen kernel reconstructed slices. ROI placement was executed in a blinded manner to the clinical features. For each ROI, gray‐level relative normalization was implemented, using the limitation of dynamics to μ ± 3 SD (gray‐level mean [μ], standard deviation [SD]) to minimize the influence of contrast and brightness variation, as described in previous studies [6, 20]. This procedure reduces possible scanner and image reconstruction variances of the CT texture features.

The following features were extracted: first order texture parameters—histogram parameters: mean value, variance, skewness, kurtosis, 1%, 10%, 50%, 90%, and 99% percentile; second order texture parameters: run‐length matrix‐based parameters (run length nonuniformity, gray‐level nonuniformity, long run emphasis, short run emphasis, and fraction of image in runs) and co‐occurrence matrix‐based parameters (angular second moment, contrast, correlation, sum of squares, inverse difference moment, sum average, sum variance, sum entropy, entropy, difference variance, and difference entropy). A total amount of 276 texture features were retrieved from every tumor. After extraction of the features, a feature reduction was performed with a Spearman's correlation analysis. Highly correlated features with an *r*‐value over 0.7 were excluded from the analysis.

**FIGURE 1:**
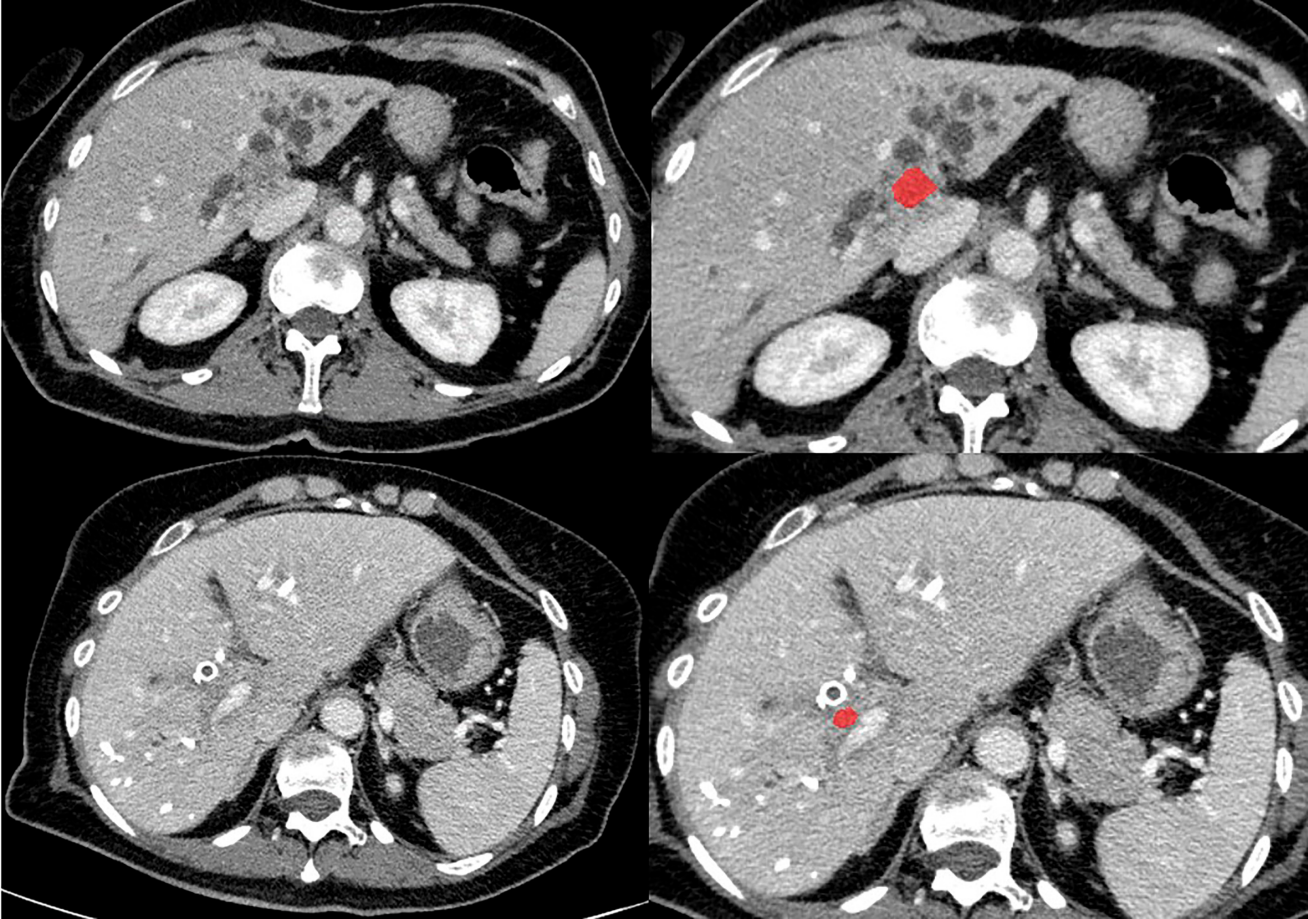
Representative cases of the patient sample. First row: 69‐year‐old female patient with long‐term survival: PT4 N0 M0, Bismuth IV, preoperative tumor markers: CEA: 1.58, CA 19‐9: 139, AFP: 3.03. Second row: 66‐year‐old female patient without long‐term survival: PT2a N0 M0, Bismuth IV, preoperative tumor markers: CEA: 1.31, CA 19‐9: 269, AFP: 3.04.

Figure 1 displays representative cases of the patient sample.

### 2.6 Statistical Analysis

Statistical analysis was performed using SPSS Statistics version 29 (IBM, Armonk, New York, USA). Collected data were evaluated by means of descriptive statistics (absolute and relative frequencies). Spearman's correlation coefficient (*r*) was used to analyze associations between texture features and grading, TNM, and tumor markers. It was used after testing for normal distribution with Kolmogorow‐Smirnow test. Spearman's correlation can be used in cases with nonnormal distribution. Differences between survival and nonsurvival were investigated by two‐tailed Mann–Whitney test in cases with nonnormal distribution.

In all instances, *p*‐values <0.05 were taken to indicate statistical significance.

## 3 Results

An overview of the investigated patient sample is provided by Table 1. Patients with long‐time survival were followed‐up without signs of tumor recurrence for a mean time of 33.3 ± 20.3 months (ranging from 8 to 61 months). In short, most cases were stage T2 (75%) and nodal negative in 13 cases (65%). During the follow‐up period, 10 patients died (45.4%).

**TABLE 1:** Overview of the descriptive statistics of the included patient sample.

	Survivors (*n* = 10)	Nonsurvivors (*n* = 12)	*p*
Gender (female, %)	5 (50)	5 (42)	0.77
Age, years	71.90 ± 7.40	71.67 ± 10.07	0.87
Bismuth classification			0.82
I	0	0	
II	0	1	
IIIA	1	0	
IIIB	0	1	
IV	9	10	
Grading			
I	0	1	
II	8	9	
III	2	2	
pT			0.08
pT1a	0	1	
pT1b	0	2	
pT2a	2	5	
pT2b	6	2	
pT3	1	1	
pT4	1	1	
N			0.38
N0	5	8	
N1	3	4	
N2	2	0	
M			0.77
M0	10	11	
M1	0	1	
Preoperative bilirubine (μmol/L)	11.1 ± 3.1	34.5 ± 16.7	**0.03**
Preoperative prothrombin time (%)	86.5 ± 20.5	86.8 ± 16.6	0.92
Preoperative CEA (μg/L)	1.9 ± 0.4	4.6 ± 5.9	0.83
Preoperative CA 19‐9 (μ/mL)	111.5 ± 38.8	221.1 ± 163.4	0.66
Preoperative AFP (μg/L)	2.4 ± 0.8	10.2 ± 17.7	0.29

*Note:* Bold indicates significant values.

### 3.1 Discrimination Analysis of Survival

In discrimination analysis for clinical outcome, the following CT texture features were statistically different between 30‐day mortality and survival: S(0,1)SumOfSqs (30‐day survival: 108.7 ± 3.9 vs. nonsurvival: 103.0 ± 3.0, *p* = 0.04); S(1,1)SumOfSqs (109.1 ± 4.0 vs. 102.1 ± 1.9, *p* = 0.02); S(1,1)InvDfMom (0.1 ± 0.02 vs. 0.1 ± 0.01, *p* = 0.04); S(4,0)Contrast (220.5 ± 27.5 vs. 182.1 ± 4.8, *p* = 0.04); S(5,0)SumOfSqs (110.3 ± 6.1 vs. 98.9 ± 1.2, *p* = 0.01); and S(5,0)InvDfMom (0.07 ± 0.02 vs. 0.12 ± 0.03, *p* = 0.04).

**FIGURE 2:**
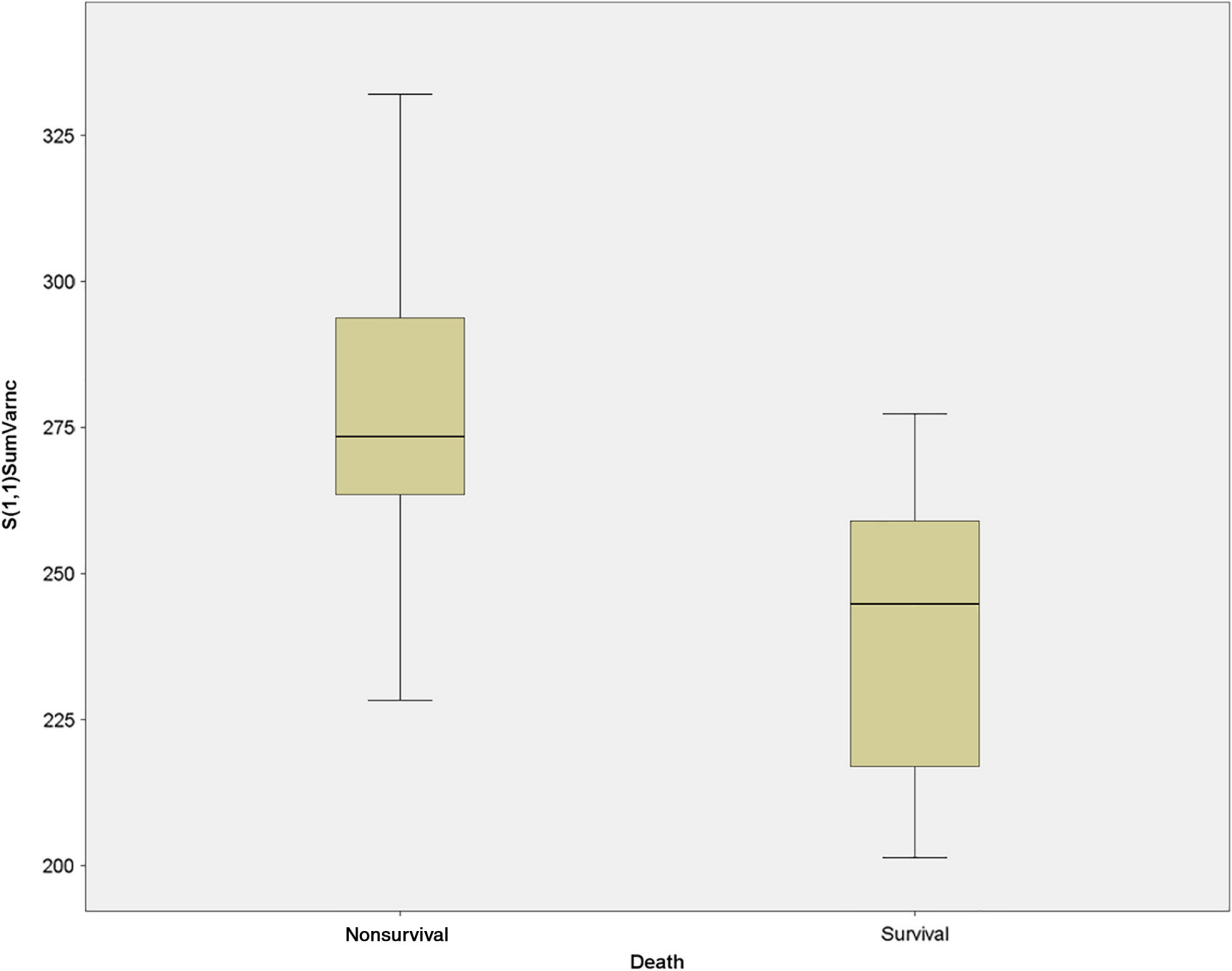
The texture parameter S(1,1)SumVarnc in comparison between long‐term survival and nonlong‐term survival (275.8 ± 32.6 vs. 239.7 ± 26.0, *p* = 0.01).

Regarding long‐term survival, the following texture parameters showed statistical significances: S(1,1)Contrast (survival: 160.8 ± 32.3 vs. nonsurvival: 191. 6 ± 22.8, *p* = 0.02); S(1,1)SumOfSqs (109.2 ± 3.2 vs. 107.8 ± 5.2, *p* = 0.01); S(1,1)SumVarnc (275.8 ± 32.6 vs. 239.7 ± 26.0, *p* = 0.01) (as shown in Figure 2); S(1,1)DifVarnc (58.2 ± 11.9 vs. 71.5 ± 12.3, *p* = 0.04); S(2,2)Correlat (0.12 ± 0.15 vs. 0.002 ± 0.07, *p* = 0.03); S(2,2)SumOfSqs (111.0 ± 4.4 vs. 105.1 ± 5.5, *p* = 0.01); S(2,2)SumVarnc (247.8 ± 32.1 vs. 210.4 ± 13.2, *p* < 0.01); S(0,4)InvDfMom (0.08 ± 0.02 vs. 0.07 ± 0.02, *p* = 0.04); S(0,4)DifVarnc (85.5 ± 17.1 vs. 70.3 ± 13.7, *p* = 0.04); S(4,4)SumOfSqs (95.3 ± 17.5 vs. 109.7 ± 8.7, *p* = 0.01); S(5,5)SumOfSqs (86.7 ± 32.6 vs. 110.2 ± 13.5, *p* = 0.03); S(5,5)SumAverg (62.3 ± 3.1 vs. 66.0 ± 3.6, *p* = 0.02); S(5,5)DifVarnc (63.0 ± 27.6 vs. 96.7 ± 24.2, *p* = 0.01); and Teta2 (−0.3 ± 0.1 vs. −0.2 ± 0.1, *p* = 0.01).

In short, several CT texture features of the second order group demonstrated the ability to discriminate between survivors and nonsurvivors.

### 3.2 Correlation With Tumor Markers

There were moderate statistical significant correlations between several texture features with the tumor marker CEA with Spearman's correlation coefficients of 0.5 and above (Table 2):

**TABLE 2:** Correlations of texture parameters with the tumor marker CEA with Spearman's correlation coefficients of 0.5 and above.

Texture parameter	Spearman's *r*	*p*
Skewness	−0.7	0.01
S(0,1)DifEntrp	−0.6	0.03
S(0,2)DifEntrp	−0.7	0.02
S(2,−2)DifVarnc	−0.6	0.04
S(3,3)Correlat	0.6	0.04
S(3,3)SumVarnc	0.6	0.04
S(4,−4)InvDfMom	−0.6	0.04
S(5,5)Contrast	0.6	0.04
S(5,5)Correlat	−0.6	0.03
S(5,5)DifVarnc	0.6	0.02
WavEnLH_s‐1	−0.7	0.01
WavEnHL_s‐2	0.7	<0.01

Skewness (*r* = −0.7, *p* = 0.01); S(0,1)DifEntrp (*r* = −0.6, *p* = 0.03); S(0,2)DifEntrp (*r* = −0.7, *p* = 0.02); S(2,−2)DifVarnc (*r* = −0.6, *p* = 0.04); S(3,3)Correlat (*r* = 0.6, *p* = 0.04); S(3,3)SumVarnc (*r* = 0.6, *p* = 0.04); S(4,−4)InvDfMom (*r* = −0.6, *p* = 0.04); S(5,5)Contrast (*r* = 0.6, *p* = 0.04); S(5,5)Correlat (*r* = −0.6, *p* = 0.03); S(5,5)DifVarnc (*r* = 0.6, *p* = 0.02); WavEnLH_s‐1 (*r* = −0.7, *p* = 0.01); and WavEnHL_s‐2 (*r* = 0.7, *p* < 0.01).

The tumor marker CA 19‐9 showed statistical significant moderate to good correlations for the following texture parameters with Spearman's correlation coefficients of 0.5 and above (Table 3):

**TABLE 3:** Correlations of the tumor marker CA 19‐9 with texture parameters resulting in Spearman's correlation coefficients of 0.5 and above.

Texture parameter	Spearman's *r*	*p*
_MinNorm	0.5	0.04
S(0,1)InvDfMom	−0.7	0.01
S(1,−1)SumVarnc	0.6	0.01
S(1,−1)DifVarnc	0.6	0.03
S(1,−1)DifEntrp	0.6	0.03
S(3,0)SumAverg	0.5	0.04
Vertl_LngREmph	−0.6	0.04
Sigma	0.7	0.01

**FIGURE 3:**
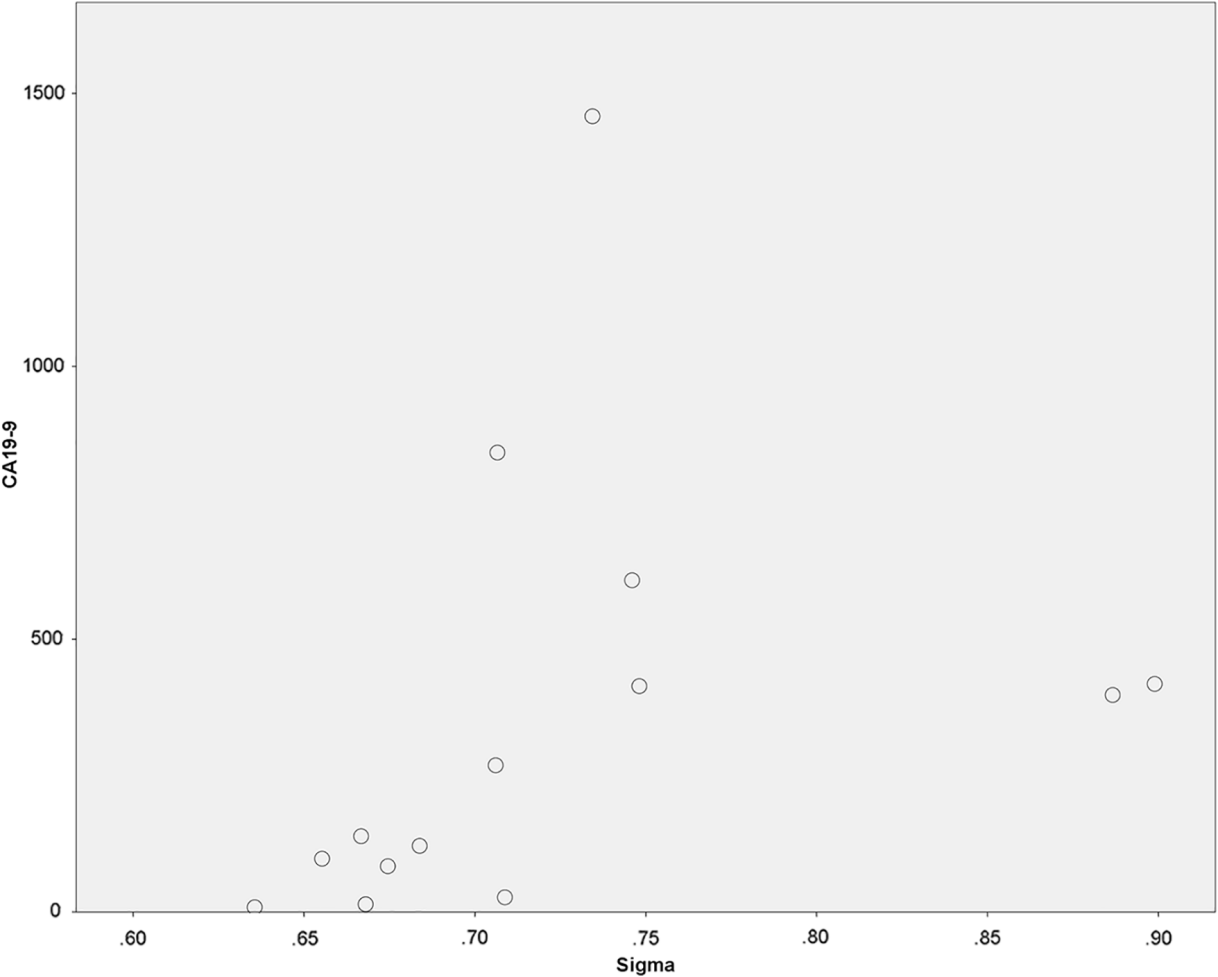
Spearman's correlation analysis between the tumor marker CA 19‐9 with the texture feature “Sigma” (*r* = 0.7, *p* = 0.01).

_MinNorm (*r* = 0.5, *p* = 0.04); S(0,1)InvDfMom (*r* = −0.7, *p* = 0.01); S(1,−1)SumVarnc (*r* = −0.6, *p* = 0.01); S(1,−1)DifVarnc (*r* = 0.6, *p* = 0.03); S(1,−1)DifEntrp (*r* = 0.6, *p* = 0.03); S(3,0)SumAverg (*r* = 0.5, *p* = 0.04); Vertl_LngREmph (*r* = −0.6, *p* = 0.04); and Sigma (*r* = 0.7, *p* = 0.01, as shown in Figure 3).

There were strong statistically significant correlations between texture features and the tumor marker AFP with Spearman's correlation coefficients of 0.75 and above:

S(0,1)AngScMom (*r* = 0.9, *p* = 0.005); S(0,1)Entropy (*r* = −0.8, *p* = 0.01); S(2,0)Entropy (*r* = −0.8, *p* = 0.03); S(0,2)AngScMom (*r* = 0.8, *p* = 0.01); S(2,−2)AngScMom (*r* = 0. 8, *p* = 0.02); S(0,4)SumVarnc (*r* = 0.8, *p* = 0.02); S(4,4)AngScMom (*r* = 0.8, *p* = 0.03); S(4,4)DifVarnc (*r* = 0.8, *p* = 0.03); S(4,−4)AngScMom (*r* = 0.8, *p* = 0.02); S(5,−5)SumOfSqs (*r* = −0.8, *p* = 0.02).

In short, CT texture features of the second order group demonstrated the strongest association with the investigated tumor markers.

### 3.3 Correlation With Tumor Grading

Correlation of texture parameters with tumor grading showed statistical significant correlation with correlation coefficients of 0.5 and above for:

S(0,1)DifEntrp (*r* = 0.5, *p* = 0.01); S(0,2)InvDfMom (*r* = −0.6, *p* = 0.01); S(3,0)DifVarnc (*r* = 0.6, *p* = 0.01); S(4,0)Contrast (*r* = 0.5, *p* = 0.02); S(4,0)DifVarnc (*r* = 0.6, *p* = 0.01); S(5,0)SumVarnc (*r* = −0.6, *p* < 0.01); and S(5,−5)SumAverg (*r* = 0.5, *p* = 0.01).

### 3.4 Correlation With TNM Stage

The following texture parameters showed statistical significant correlations with T stage with correlation coefficients of 0.5 or above: S(0,5)Contrast (*r* = 0.5, *p* = 0.01) and S(5,5)DifVarnc (*r* = −0.5, *p* = 0.02).

Regarding the N stage texture parameters two correlations were identified, S(4,−4)Contrast (*r* = 0.5, *p* = 0.01) and S(4,−4)SumOfSqs (*r* = 0.6, *p* = 0.01). There were no statistically significant associations with positive M status.

## 4 Discussion

The present study identified statistically significant associations between texture features derived from CT images of perihilar cholangiocarcinomas with the patient survival. Another key finding is that some texture features showed correlations with grading and T/N‐stadium. These findings highlight the possible importance of CT texture analysis to better characterize these kinds of tumors in a noninvasive way. The observed association with the further clinical outcome after surgical treatment might heavily affect clinical management in the future. Interestingly, there were also significant correlations of CT texture parameters with the widely used tumor markers CA‐19‐9, CEA, and AFP.

The morphological CT imaging features of extrahepatic perihilar cholangiocarcinoma are usually presenting with low attenuation pre‐contrast and tend to show contrast‐enhancement in the portal venous phase. Sometimes, especially intraductal lesions are not presenting as any visible mass and can be diagnostic challenging. In those cases, presumably CT texture analysis could also aid in discrimination purposes. Commonly, proximal dilatation of the bile ducts is present and helps delineating the lesions [21]. The clinical outcome of the patients differs significantly according to resectability [22]. Furthermore, poor tumor differentiation has been shown to be a viable predictor of patient long‐time survival, a factor that is only poorly reflected by conventional CT‐imaging. Also, lymph node involvement is an important factor as a further independent predictor of the outcome [23]. At present, the widely used TNM staging system and also the Mayo staging system allow preoperative patient prognosis assessment, although the analysis of their prognostic capabilities has only been reported with areas under the curve of 0.50 and 0.59, respectively [24]. Therefore, there is a definite need for better characterization of these patients as well as the establishment of novel prognostic biomarkers.

The present study only investigated surgically treated patients. If the radiologists could predict the underlying tumor aggressiveness of the tumor by presurgical imaging, this could improve diagnostic power in the clinical routine as well as potentially reduce invasive procedures. The present results suggest that a promising parameter could be the advanced entropy‐based texture feature “SumVarnc,” which might aid in the prediction of patient outcome. This might be accounted for by the well‐known possibility of that entropy parameter to quantify the heterogeneity of the tumors, which has been shown to be associated with underlying histopathology features [3].

Our findings are in line with a recent study on the same topic, which has been showing the usefulness of radiomics in building multivariate prediction models for early recurrence in a small patient collective [25]. Another work group performed a radiomics study on presurgical MRI and found that a combined clinical and radiomics‐based nomogram performed significantly better in the prediction of 1‐, 3‐ and 5‐year survival than the classical TNM‐staging system [24]. When comparing the different nomograms, the combined clinical and radiomic approach performed only slightly better than the radiomic nomogram, although with no statistically significant difference. These results are very promising and could prove very helpful in future clinical care employing CT and MRI images [26, 27].

Furthermore, a recent PET/CT‐study showed the ability of texture analysis to predict pathology data and patient outcome in a cohort with intrahepatic cholangiocarcinoma. Interestingly, in this study, it was also shown that also the radiomics of the peritumoral areas had clinical relevance [28]. This should be emphasized that not only the texture features of the tumor itself but also the peritumoral region might be of prognostic relevance.

It has to been taken in account though that the stability of texture features between different scanners and reconstruction methods was investigated in many previous studies and is still a challenge for possible future clinical translation [29]. Furthermore, the different lesion size has to be considered as a possible factor influencing texture features, as already has been shown in phantom studies [30]. Also, the way of contrast media application has to been taken account for, as a recent study has been showing possible differences in some texture parameters between native and contrast enhanced CT scans [31]. Yet, in the present analysis, only CT images in portal venous phase were included to reduce heterogeneity. However, texture features derived from CT images are in general considered more reliable compared with texture features from MRI [32]. Another recent study investigated the reproducibility of texture features between different segmentations by different operators and also different contrast phases, which could prove a reproducibility ranging from 67%–100% [33] Naturally, also besides the mentioned challenges of texture analysis in clinical translation, our study is not without some further limitations. A further important aspect is the need to acknowledge interscanner variability between different vendors and CT scanner generations.

Beyond the inherent limitations of CT texture analysis, the present study has some limitations to address. As a retrospective study, there is a possible known inherent bias. Yet, this was accounted for by blinded reading of the CT to clinical features. Furthermore, as a single center study, the patient sample is rather small. Factors such as slight differences in contrast media application, lesion size, and localization could have been influencing the texture features analysis. Another important limitation is a possible interreader heterogeneity influenced by the ROI measurement. This was accounted for by the consulting of a senior radiologist to define the tumor boundary. However, some bias induced by the measurement may still be present.

The limitations need to be addressed by further studies, most promising in a multicenter design to validate the possible clinical benefit of CT texture analysis in patients with perihilar cholangiocarcinoma. Nevertheless, the present analysis demonstrates the possible prognostic relevance of the CT texture analysis in these patients.

In conclusion, several texture features derived from CT images were associated with survival, tumor stage, and tumor markers in patients with perihilar cholangiocarcinoma. Therefore, CT texture features could be used as promising novel imaging markers in patients with perihilar cholangiocarcinoma.

## Author Contributions

**Jakob Leonhardi:** data curation (equal), formal analysis (equal), investigation (equal), methodology (equal), writing – original draft (equal). **Arsen Sabanov:** data curation (equal), investigation (equal), resources (equal). **Anne Kathrin Höhn:** data curation (equal), resources (equal), writing – review and editing (equal). **Robert Sucher:** resources (equal), supervision (equal), writing – review and editing (equal). **Daniel Seehofer:** resources (equal), supervision (equal), writing – review and editing (equal). **Matthias Mehdorn:** resources (equal), supervision (equal), writing – review and editing (equal). **Benedikt Schnarkowski:** data curation (equal), formal analysis (equal), investigation (equal). **Sebastian Ebel:** resources (equal), supervision (equal), writing – review and editing (equal). **Timm Denecke:** resources (equal), supervision (equal), writing – review and editing (equal). **Hans‐Jonas Meyer:** methodology (equal), project administration (equal), resources (equal), software (equal), supervision (equal), validation (equal), visualization (equal), writing – original draft (equal), writing – review and editing (equal).

## Ethics Statement

For this retrospective, observational study, all procedures performed involving human participants were in accordance with the ethical standards of the institutional and/or national research committee and with the 1964 Helsinki declaration and its later amendments or comparable ethical standards. The study was approved by the local ethical commission board from the University of Leipzig (AZ‐ EK: 243–14‐14 072 014).

## Consent

All participants provided written informed consent of this post hoc scientific analysis and the publication.

## Conflicts of Interest

The authors declare no conflicts of interest.

## Data Availability

Data available on request due to privacy/ethical restrictions.

## References

[1] M. Incoronato , M. Aiello , T. Infante , et al., “Radiogenomic Analysis of Oncological Data: A Technical Survey,” International Journal of Molecular Sciences 18, no. 4 (2017): 805. 28417933 10.3390/ijms18040805 PMC5412389

[2] G. Wu , A. Jochems , T. Refaee , et al., “Structural and Functional Radiomics for Lung Cancer,” European Journal of Nuclear Medicine and Molecular Imaging 48, no. 12 (2021): 3961–3974. 33693966 10.1007/s00259-021-05242-1 PMC8484174

[3] N. Just , “Improving Tumour Heterogeneity MRI Assessment With Histograms,” British Journal of Cancer 111, no. 12 (2014): 2205–2213. 25268373 10.1038/bjc.2014.512 PMC4264439

[4] H. J. Meyer , J. Leonhardi , A. K. Höhn , et al., “CT Texture Analysis of Pulmonary Neuroendocrine Tumors—Associations With Tumor Grading and Proliferation,” Journal of Clinical Medicine 10, no. 23 (2021): 5571. 34884272 10.3390/jcm10235571 PMC8658090

[5] H. J. Meyer , G. Hamerla , A. K. Höhn , and A. Surov , “CT Texture Analysis—Correlations With Histopathology Parameters in Head and Neck Squamous Cell Carcinomas,” Frontiers in Oncology 9 (2019): 444. 31192138 10.3389/fonc.2019.00444 PMC6546809

[6] H. J. Meyer , S. Schob , A. K. Höhn , and A. Surov , “MRI Texture Analysis Reflects Histopathology Parameters in Thyroid Cancer—A First Preliminary Study,” Translational Oncology 10, no. 6 (2017): 911–916. 28987630 10.1016/j.tranon.2017.09.003 PMC5645305

[7] L. G. T. Morris , N. Riaz , A. Desrichard , et al., “Pan‐Cancer Analysis of Intratumor Heterogeneity as a Prognostic Determinant of Survival,” Oncotarget 7, no. 9 (2016): 10051–10063. 26840267 10.18632/oncotarget.7067 PMC4891103

[8] A. Al Mahjoub , V. Bouvier , B. Menahem , et al., “Epidemiology of Intrahepatic, Perihilar, and Distal Cholangiocarcinoma in the French Population,” European Journal of Gastroenterology & Hepatology 31, no. 6 (2019): 678–684. 30633038 10.1097/MEG.0000000000001337

[9] B. Blechacz , M. Komuta , T. Roskams , and G. J. Gores , “Clinical Diagnosis and Staging of Cholangiocarcinoma,” Nature Reviews. Gastroenterology & Hepatology 8, no. 9 (2011): 512–522. 21808282 10.1038/nrgastro.2011.131 PMC3331791

[10] C. Song , K. Kim , E. K. Chie , et al., “Nomogram Prediction of Survival and Recurrence in Patients With Extrahepatic Bile Duct Cancer Undergoing Curative Resection Followed by Adjuvant Chemoradiation Therapy,” International Journal of Radiation Oncology, Biology, Physics 87, no. 3 (2013): 499–504. 24074923 10.1016/j.ijrobp.2013.06.2041

[11] B. Groot Koerkamp , J. K. Wiggers , M. Gonen , et al., “Survival After Resection of Perihilar Cholangiocarcinoma—Development and External Validation of a Prognostic Nomogram,” Annals of Oncology 26, no. 9 (2015): 1930–1935. 26133967 10.1093/annonc/mdv279 PMC4754626

[12] H. W. Chen , A. Z. Pan , Z. J. Zhen , et al., “Preoperative Evaluation of Resectability of Klatskin Tumor With 16‐MDCT Angiography and Cholangiography,” AJR. American Journal of Roentgenology 186, no. 6 (2006): 1580–1586. 16714646 10.2214/AJR.05.0008

[13] H. Y. Lee , S. H. Kim , J. M. Lee , et al., “Preoperative Assessment of Resectability of Hepatic Hilar Cholangiocarcinoma: Combined CT and Cholangiography With Revised Criteria,” Radiology 239, no. 1 (2006): 113–121. 16467211 10.1148/radiol.2383050419

[14] I. Joo , J. M. Lee , and J. H. Yoon , “Imaging Diagnosis of Intrahepatic and Perihilar Cholangiocarcinoma: Recent Advances and Challenges,” Radiology 288, no. 1 (2018): 7–13. 29869969 10.1148/radiol.2018171187

[15] S. M. Strasberg , “Nomenclature of Hepatic Anatomy and Resections: A Review of the Brisbane 2000 System,” Journal of Hepato‐Biliary‐Pancreatic Surgery 12, no. 5 (2005): 351–355. 16258801 10.1007/s00534-005-0999-7

[16] H. M. Hau , M. Devantier , N. Jahn , et al., “Impact of Body Mass Index on Tumor Recurrence in Patients Undergoing Liver Resection for Perihilar Cholangiocarcinoma (pCCA),” Cancers (Basel) 13, no. 19 (2021): 4772. 34638257 10.3390/cancers13194772 PMC8507532

[17] M. Shimada , Y. Yamashita , S. Aishima , K. Shirabe , K. Takenaka , and K. Sugimachi , “Value of Lymph Node Dissection During Resection of Intrahepatic Cholangiocarcinoma,” British Journal of Surgery 88, no. 11 (2002): 1463–1466. 10.1046/j.0007-1323.2001.01879.x 11683741

[18] M. Strzelecki , P. Szczypinski , A. Materka , and A. Klepaczko , “A Software Tool for Automatic Classification and Segmentation of 2D/3D Medical Images,” Nuclear Instruments and Methods in Physics Research 702 (2013): 137–140.

[19] P. M. Szczypiński , M. Strzelecki , A. Materka , and A. Klepaczko , “MaZda—A Software Package for Image Texture Analysis,” Computer Methods and Programs in Biomedicine 94, no. 1 (2009): 66–76. 18922598 10.1016/j.cmpb.2008.08.005

[20] J. Fruehwald‐Pallamar , C. Czerny , L. Holzer‐Fruehwald , et al., “Texture‐Based and Diffusion‐Weighted Discrimination of Parotid Gland Lesions on MR Images at 3.0 Tesla,” NMR in Biomedicine 26, no. 11 (2013): 1372–1379. 23703801 10.1002/nbm.2962

[21] Y. E. Chung , M. J. Kim , Y. N. Park , et al., “Varying Appearances of Cholangiocarcinoma: Radiologic‐Pathologic Correlation,” Radiographics 29, no. 3 (2009): 683–700. 19448110 10.1148/rg.293085729

[22] G. Spolverato , M. Y. Yakoob , Y. Kim , et al., “The Impact of Surgical Margin Status on Long‐Term Outcome After Resection for Intrahepatic Cholangiocarcinoma,” Annals of Surgical Oncology 22, no. 12 (2015): 4020–4028. 25762481 10.1245/s10434-015-4472-9

[23] H. M. Hau , F. Meyer , N. Jahn , S. Rademacher , R. Sucher , and D. Seehofer , “Prognostic Relevance of the Eighth Edition of TNM Classification for Resected Perihilar Cholangiocarcinoma,” Journal of Clinical Medicine 9, no. 10 (2020): 3152. 33003424 10.3390/jcm9103152 PMC7599593

[24] Z. Sun , X. Sun , J. Guo , et al., “Prognostic Influence for Hilar Cholangiocarcinoma and Comparisons of Prognostic Values of Mayo Staging and TNM Staging Systems,” Medicine 101, no. 49 (2022): e32250. 36626512 10.1097/MD.0000000000032250 PMC9750704

[25] H. Qin , X. Hu , J. Zhang , et al., “Machine‐Learning Radiomics to Predict Early Recurrence in Perihilar Cholangiocarcinoma After Curative Resection,” Liver International 41, no. 4 (2021): 837–850. 33306240 10.1111/liv.14763

[26] J. Zhao , W. Zhang , C. L. Fan , et al., “Development and Validation of Preoperative Magnetic Resonance Imaging‐Based Survival Predictive Nomograms for Patients With Perihilar Cholangiocarcinoma After Radical Resection: A Pilot Study,” European Journal of Radiology 138 (2021): 109631. 33711571 10.1016/j.ejrad.2021.109631

[27] J. Zhao , W. Zhang , Y. Zhu , et al., “Development and Validation of Noninvasive MRI‐Based Signature for Preoperative Prediction of Early Recurrence in Perihilar Cholangiocarcinoma,” Journal of Magnetic Resonance Imaging 55, no. 3 (2022): 787–802. 34296802 10.1002/jmri.27846

[28] F. Fiz , C. Masci , G. Costa , et al., “PET/CT‐Based Radiomics of Mass‐Forming Intrahepatic Cholangiocarcinoma Improves Prediction of Pathology Data and Survival,” European Journal of Nuclear Medicine and Molecular Imaging 49, no. 10 (2022): 3387–3400. 35347437 10.1007/s00259-022-05765-1

[29] D. Mackin , X. Fave , L. Zhang , et al., “Measuring Computed Tomography Scanner Variability of Radiomics Features,” Investigative Radiology 50, no. 11 (2015): 757–765. 26115366 10.1097/RLI.0000000000000180 PMC4598251

[30] L. J. Jensen , D. Kim , T. Elgeti , I. G. Steffen , B. Hamm , and S. N. Nagel , “Stability of Radiomic Features Across Different Region of Interest Sizes—A CT and MR Phantom Study,” Tomography 7, no. 2 (2021): 238–252. 34201012 10.3390/tomography7020022 PMC8293351

[31] R. Kakino , M. Nakamura , T. Mitsuyoshi , et al., “Comparison of Radiomic Features in Diagnostic CT Images With and Without Contrast Enhancement in the Delayed Phase for NSCLC Patients,” Physica Medica 69 (2020): 176–182. 31918370 10.1016/j.ejmp.2019.12.019

[32] N. Rekhtman , “Lung Neuroendocrine Neoplasms: Recent Progress and Persistent Challenges,” Modern Pathology 35 (2022): 36–50. 34663914 10.1038/s41379-021-00943-2 PMC8695375

[33] I. S. Gruzdev , K. A. Zamyatina , V. S. Tikhonova , et al., “Reproducibility of CT Texture Features of Pancreatic Neuroendocrine Neoplasms,” European Journal of Radiology 133 (2020): 109371. 33126173 10.1016/j.ejrad.2020.109371

